# UK Health Workers’ Experiences of Striking and Not Striking in the 2022–2024 Industrial Disputes

**DOI:** 10.1155/jonm/3663164

**Published:** 2026-02-26

**Authors:** Ryan Essex, David Smithard

**Affiliations:** ^1^ The George Institute for Global Health, Sydney, Australia; ^2^ School of Population Health, University of New South Wales, Sydney, Australia, unsw.edu.au; ^3^ Institute for Lifecourse Development, University of Greenwich, London, UK, gre.ac.uk; ^4^ Elderly Care, Queen Elizabeth Hospital, Lewisham and Greenwich NHS Trust, London, UK, nhs.uk

**Keywords:** healthcare, industrial dispute, nurse, physician, protest, strike, UK

## Abstract

Starting in 2022 and continuing into 2024, health workers in the UK voted to take widespread strike action. Amongst the literature that has examined healthcare strikes, there is a paucity of qualitative literature and little that explores the perspectives of those who work through strikes. This study sought to address this, exploring the perspectives of health workers who did and did not go on strike in the United Kingdom throughout the 2022–2024 disputes. Semistructured interviews were carried out with an interdisciplinary sample of NHS staff who both did and did not strike. Three themes emerged: *reasons for (not) striking, managing the strikes, and views of the NHS.* There were several factors that shaped participants’ views about and experiences of the strikes. Most participants, even those who did not strike, had sympathy for the strikes and strikers. While the impact of the strikes was not felt equally, with some staff facing fare more acute challenges, many felt that any disruption caused by the strikes was not much worse than a regular busy day in the NHS. Related to this and one point where there was substantial convergence was participants’ views of the NHS. Regardless of profession or whether they went on strike, and while all felt a great sense of pride working in the NHS, all saw the NHS as facing multiple and broad crises and the need for substantial reform.

## 1. Introduction

In late 2022, nurses in the United Kingdom took unprecedented action, with the Royal College of Nursing (RCN) balloting its members to gauge support for industrial action^1^. This was the first time the RCN had taken such action in its 106‐year history. The ballot came after a pay offer from the government, which was well below inflation. Several other unions followed suit, and while most disputes were settled in 2023, resident doctors continued striking throughout 2024 until a settlement was reached. Throughout the strikes, the UK government blamed unions and health workers. The Health Secretary at the time, Steve Barclay, accused ambulance unions of taking a “conscious choice to inflict harm on patients” [1]. At the same time, professional associations and unions released equally as pointed statements. In response to the governments’ ongoing refusal to negotiate, in 2024, the British Medical Association (BMA) accused the government of “consciously and deliberately overseeing the demise of the NHS at a point when it is needed most” [2].

The above finger pointing aside, in reality, measuring the impact of strikes is quite difficult. The evidence is mixed when it comes to patient mortality and other outcomes, with no clear association between strike action and increased mortality or other patient outcomes [3, 4]. Recent studies have attempted to overcome methodological limitations present in the broader literature by using more granular data [5]. Regardless of their impact and the difficulties in measuring some outcomes, strikes are undoubtedly disruptive, and there are consistent trends seen in the literature when it comes to healthcare delivery. That is, strikes most often result in cancelled appointments and fewer attendances to hospital [6].

Amongst the literature on healthcare strikes, we find a reasonable number of studies that have examined patient and health worker perspectives of strike action. As a whole, these studies speak to the divisive nature of strikes, the varying support they receive and the factors that predict such support. There are some notable shortcomings within this literature. Amongst 34 studies published in a 2022 scoping review, only four included views of patients and their relatives. There was also a paucity of qualitative studies (*n* = 10) and studies that included both those who went on strike and those who did not [4]. This study sought to address this, by adding to the qualitative evidence on strike action, including the perspectives of those who both went on strike and those who did not. Thus, the aim of this study was to explore the perspectives of health workers who did and did not go on strike in the United Kingdom throughout the 2022–2024 industrial disputes. This study sought to add important context and nuance to the quantitative literature on strikes, providing insights into how strikes are managed, mitigated and felt by staff, amongst other things.

## 2. Methods

### 2.1. Procedure and Participants

This study recruited healthcare workers based in the United Kingdom. Participants were initially contacted through the personal networks of the authors; we also utilised snowball sampling. All health workers were eligible, as long as they were employed at the time of the strikes. All worked in NHS trusts throughout England and primarily in London. Participants contacted the researchers if they were interested in participating in the study and arranged a time for an interview. Interviews were conducted throughout late 2023 to mid 2024, while the consultant and resident doctor strikes were ongoing. Interviews were semistructured and carried out by RE. All interviews lasted between 30 and 60 min. An interview schedule was developed in line with the existing literature on strike action (see supporting information), with questions that explored experiences of and views toward the strikes, alongside the impacts the strikes had. More broadly, the interviews also explored participants’ views about the current state and future of the NHS. Interviews were audio recorded and transcribed.

### 2.2. Analysis

To analyse participant transcripts, reflexive thematic analysis was employed [8, 9]. This approach has been widely utilised in healthcare research. Reflexive thematic analysis offers a flexible yet systematic approach to qualitative analysis. Generally, this type of analysis is carried out in six phases, and each will be reported below, along with how each step was applied in this study. (1) Data familiarisation. After transcription, data were read and re‐read with each of the researchers familiarising themselves with each transcript and the overall dataset. (2) Coding. Initial codes were then generated by RE. (3) Generating initial themes. Codes were then collated into initial themes. This involved a process of organising codes under broader categories. (4) Developing and reviewing themes. After data were collated preliminary themes were reviewed and where needed, recategorised. This was done several times, until we were happy that there was a coherent meaning/concept/idea underlying each theme. (5) Refining, defining, and naming themes. All authors contributed to the further refining and naming of themes. (6) Writing up. RE led the initial write up of the paper. This was reviewed and subsequently revised by all named authors. As is common in reflexive thematic analysis, we noted some conceptual overlap between themes. Rather than treating this as a problem of rigour, we approached overlap as reflecting the complexity of participants’ accounts and the fact that some ideas cut across multiple domains. Accordingly, data extracts were coded to more than one code where appropriate, and themes were refined iteratively to ensure each theme captured a coherent central organising concept, with any related subthemes used only to structure distinct facets within that concept. The below results are reported in line with standards for reporting qualitative research (SRQR) guidance [10].

### 2.3. Ethical Approval

Ethical approval for this study was granted by the University of Greenwich′s Research Ethics Board (23.1.5.3)^2^. Written consent was obtained from all participants prior to commencing each interview, and all participants were provided with the opportunity to withdraw from the study, both before and during the interview. NHS ethical approval was not required for this work as all participants were healthcare workers, who recruited because of their professional roles (see [11])

## 3. Results

The results below include eight participants, six who did not strike and two who did strike. Participants included three resident doctors, two consultants, two physician associates (PAs) and one advanced clinical practitioner (ACP). Analysis revealed three overarching themes, ‘reasons for (not) striking’, ‘managing the strikes’ and ‘views of the NHS’. Each had a number of subthemes, which are outlined in the following.

### 3.1. Reasons for (Not) Striking

The issue that dominated most discussion related to participants reasons for either striking or not striking. Participants sat across a spectrum in how strongly they felt about whether the strikes could be justified. While there was a small number of participants who felt strongly about striking (or not), most felt quite divided on whether striking was or was not the right thing to do, often citing very specific reasons why they did or did not strike. At the same time, others expressed solidarity or understanding with the strikes, even when not going on strike themselves. There were a range of positions and reasons given in relation to participants’ reasons for (not) striking, and these fell into three subthemes labelled: ‘conduct and demands of the strikes’, ‘personal reasons for (not) striking’ and ‘risks to patients’. Each will be discussed below.

#### 3.1.1. Conduct and Demands of the Strikes

One issue that dominated discussions had to do with the conduct and demands of the strikes. That is, participants discussed why they felt there was a need (or not) for the strikes and whether they felt these reasons were justified, along with offering reflections on the way that the strikes were conducted.

A major point of concern, regardless of whether participants went on strike or not had to do with pay and conditions within the NHS. Some felt that there were noticeable changes in pay and conditions that had occurred during their time in training, with a number reflecting on the fact that pay had not increased and conditions had deteriorated since they started in the NHS. For some, this made striking a fairly easy decision. One participant spoke about the impact that pay had on retention of staff and how a strike could be used to signal discontent with the government and its management of the NHS. In saying this, most participants who did not strike raised concerns about the dispute as it was primarily about pay, the decision whether to strike or not however was not taken lightly. One participant, who went on strike in the resident doctor strikes of 2015/2016, explained why they were uncomfortable in striking in the more recent disputes:

*“In the 2015, 2016 junior doctor strikes, I was really in favour of them and I did take part in the strikes. And I encouraged people to take part in strikes and I was very, very vocal. Because at that time it felt like we were striking against something that was unsafe and unfair… Whereas this time round, it was straight away into a full walk out and only about pay* (P5, resident doctor, non‐striker)”

One participant who was strongly against the strike spoke about how uncomfortable they were about striking only for pay, noting how much power the professions had and that this was being used in a *“very* aggressive *way to get more money for ourselves”* (P4, consultant, nonstriker). This participant went on to explain that it was not only that the strikes did not make broader demands about the state of the NHS and the difficulties it continued to face but also that doctors (both resident and consultant) were already amongst the best paid staff within the NHS.

For others, when it came to pay, it mattered who went on strike. As was alluded to by the participant above, in considering how NHS funding is distributed amongst staff, even if opposed to a strike by their own professional group, a number of participants could understand why other staff were striking. Importantly, this was not just about other staff members who were paid less; both an ACP and PA who did not strike expressed sympathy with the resident doctors’ strikes for example.

Beyond the reasons for striking, participants also expressed concerns with the conduct of the strikes, that is, the length of the strikes, when they were undertaken, along with the fact that the demands being made (particularly by the resident doctors) were unattainable. One participant was not only uncomfortable with the demands around pay but also the fact that the strikes had escalated to increasingly longer walk outs. One participant who expressed support for the resident strikes questioned how they were being conducted, particularly around Christmas and New Year, a time where staffing is typically limited.

At the time of writing, all NHS strikes were resolved, at the time the interviews were conducted however, little progress had been made with negotiations. At least two participants questioned the fact that the strikes appeared likely to persist into the foreseeable future, questioning the concessions needed from the government and unions. Another participant, despite being supportive of the strikes, questioned whether the full pay restoration demanded by the BMA was achievable and how this may have been counter‐productive for their cause. They went on to explain how this was increasingly impacting solidarity and cooperation between consultants and residents.

#### 3.1.2. Personal Reasons for (Not) Striking

A range of other, more personal reasons were also raised as being important factors that dictated support or otherwise for the strikes. At least one participant spoke about their decision to strike as being connected to deeply personal reasons, their upbringing, with their parents involved in the trade‐union movement and having gone on a number of protests in the past. While one participant agreed with others going on strike in principle, they did not participate, mainly because of *“the cost of living going up … and having our mortgages go up”* (P2, ACP, non‐striker). A number of other participants also cited the financial strain that came with going on strike.

While not a critical factor in determining whether they went on strike or not, most participants reflected on the broader social pressures related to (not) striking and the support they had received from the public. A number of participants spoke about the pressure within their workplaces; sometimes, this was overt:

*“At one of the hospitals they put a sign up in A&E one strike day saying, ‘The waiting time is going to be really, really long because doctors are on strike.’ Obviously, the waiting time is really long anyway when we′re not on strike. But we never put a sign up to say, ‘The wait time is really long because we′re kind of an underfunded hospital and health system.’ So it does feel like they′re kind of unfairly often putting blame on doctor′s striking when there is lots of other reasons”* (P3, resident doctor, striker).

This pressure was present in other, *more* subtle ways. A resident doctor spoke about progressing through their training, and how they were at *“the point in training where I need to make a good impression on people because I want to work [as a consultant]”* (P8, resident doctor, striker). These considerations extended beyond the workplace with almost all participants commenting on the broader contention surrounding the dispute. One participant commented on the fact that not only did they believe that there was little solidarity from the public in regards to the strikes, but that this no longer mattered, particularly for the residents who went on strike: *“… but I see from my colleagues that they didn′t care about solidarity this time… They weren′t really interested in public opinion. I think that’s what has changed in the last year”* (P6, PA, non‐striker).

Importantly, whether people went on strike or not was not only shaped by feeling pressured not to strike, a number of participants (as will also be seen in the theme discussing conflict and solidarity) were encouraged by the public response to the strikes. Finally, one participant discussed the fact that they had gone part‐time a number of years earlier. Because of this, the fact they only worked part‐time, they felt that the strike had far less of an impact on them and that they were able to step back and manage the stress exacerbated by the strikes far better than their colleagues.

#### 3.1.3. Risks to Patients

Whether participants went on strike or not and regardless of their position, almost every participant mentioned the issue of patient safety and the risks that strike action presented. Even those who went on strike were mindful of the impact on patients, one participant who went on strike stressed that *“I want it [the dispute] to be resolved without having an impact felt by patients and my consultant colleagues”* (P8, resident doctor, striker). However, the extent to which patient safety was seen as important and perceptions about the actual impact of the strikes varied. One participant, who was against the strikes felt one of the primary reasons they could not be justified had to do with impact they could have on patients. One participant who was otherwise sympathetic to the strikes provided a personal story about a family member who was undergoing diagnostic tests for cancer at the time of the strikes, reflecting on the how the delays during the strike made this more stressful. For some, the conflict in whether to strike resulted in them abstaining from the strikes (for lack of a better term). One participant described how, although they did not strike and were not in full agreement with the strikes, at the same time wanted to support those who went on strike:

*“I ended up swapping my research days where I was under a different contract. So the days of the strike, I always ended up either being on annual leave or on a non NHS working day… I wanted to support the strike … But for me, at an individual personal level, I didn′t want to withhold my care”* (P5, resident doctor, non‐striker).

How the strikes were conducted mattered for when assessing risk to patients. One participant reflected on the fact that they felt nursing colleagues put greater safeguards in place during their strikes, when compared to the resident strikes. When it came to risk, others however felt it was inaccurate to define this as simply an issue that fell on health workers shoulders. They reflected on the fact that others, like the hospital and government, also held some responsibility for patient safety.

#### 3.1.4. Managing the Strikes

Participants spoke at length about the impact of the strikes and how they managed throughout when working during the strikes. For those who did not strike, how the impact of the strike was felt varied substantially, this came down to a range of factors, their position, who was on strike and the support they had from their colleagues, amongst other factors. Equally as varied were perceptions about the impact of the strikes on patient care. Some participants reported that patients had raised concerns, while others discussed how patients were unaware that the strikes were happening. Another area where the strikes had substantial impact was on teamwork and relationships with colleagues. While this created tension in a range of ways, the strikes also created pockets of solidarity, not only amongst strikers but also those working throughout the strikes, having to adapt to new working conditions. In terms of the impact and management of the strikes, four subthemes emerged: ‘workload and healthcare delivery’, ‘preparation and mitigation’, ‘conflict and solidarity in and around the strikes’ and ‘patient perceptions and impact’. Each will be discussed below.

#### 3.1.5. Workload and Healthcare Delivery

Almost all participants and particularly those who worked throughout the strikes spoke about the impact that the strikes had on their workload and not only this, how it shifted the delivery of care. A number of participants referred to the existing pressures that the NHS was already under, discussing how this shaped their experiences of the strike. While almost all acknowledged that there was *“existing strain on being underfunded and understaffed under equipped under appreciated”* (P1, PA, non‐striker), many felt this made it somewhat difficult to distinguish between the impacts of the strike and any normal day working in the NHS: *“… A&E has chaotic days, non-chaotic days and things like that, so it could have just been an average chaotic day”* (P2, ACP, non‐striker).

Others, however, provided a different perspective. A number of participants felt that the strikes had minimal impact on their roles because there was simply not that many people in their departments who went on strike. Others reflected on how staffing was prioritised for their section of the hospital, increasing the number of staff who may have normally been on shift and making their roles easier. The strikes were felt in other ways. Some felt more daunted by the shifts in their role and increased workload, while others felt they coped well with the extra responsibility that came with working through the strikes. One issue that dominated discussions was how the resident doctors’ strikes impacted the delivery of care. Some reflected on the fact that consultants had different skill sets to residents and noted how this caused issues, with things like note taking and drug charts. One consultant discussed how they adapted to this, speaking about how informal networks were formed and group chats used to support one another.

While there were challenges in adapting to the strikes, overall most who worked during the resident doctors’ strikes felt that things actually worked better or were at least more efficient. This was a common reflection amongst almost all participants. Some reflected on things being done *“quicker and smoother”* (P1, PA, nonstriker) and *“more efficient[ly]”* (P2, ACP, nonstriker) as decisions were made *“then and there, so things actually progress quite quickly”* (P6, PA, nonstriker).

#### 3.1.6. Preparation and Mitigation

Closely related to how the strikes impacted the delivery of care and workload, a number of participants also discussed the steps that were taken to prepare for and mitigate the impacts of the strikes. These steps included meetings about staffing, what services were to be prioritised and reflections from staff about what worked and what did not during previous strikes. This often started with a rota or an identification of services that were to be covered and recruiting staff to cover shifts. Participants spoke about prioritising discharges and the most unwell patients, while others also spoke about having to prioritise what got done while on shift. Some noted that as the strikes went on hospitals became more adept at managing the disruption. Participants discussed how management had gotten better at identifying and prioritising the services that were needed to ensure that hospitals continued to funciton throughout the strikes.

#### 3.1.7. Conflict and Solidarity in and Around the Strikes

The strikes also influenced cooperation and conflict between staff. This was not a simple binary between striking and nonstriking staff and, in fact, more often than not there was no open animosity. Rather, solidarity and conflict emerged in far more subtle ways between and within different groups during the strikes. A number of participants reflected on the fact that there was generally increased stress and tension between staff around the strikes. A small number of participants discussed how these tensions had occasionally come to a head, citing examples of disparaging comments from more senior staff about the strikes.

The strikes also caused disagreements between resident doctors, particularly when it came to discussions about the aims or ends of the strike. There were also examples of the strikes exacerbating tensions between consultants. One consultant was critical of colleagues who they felt had signed up for shifts to cover the resident strikes that they were not capable of doing. In turn, they only created further discord and stress for others. Some felt no changes in that they did not face any specific conflict or tension during the strike; however, most reflected on the fact that while there was tension, this was rarely expressed openly. That is, many acknowledged there were tensions and differences in opinion between staff, but these were not openly discussed.

*As well as there being tension and disagreement within and between some of the professions who were involved, the strikes also promoted solidarity. This was found between those who worked throughout the strikes. Consultants spoke about forming informal groups and group chats, where they supported one another, drawing on their specialities and expertise, particularly during night shifts. They adapted to a new way of working, with many expressing how willing colleagues were to assist during this time. Solidarity was also found elsewhere, one participants explained how the issues surrounding the strike had galvanised them and other residents: “… I remember when the results came out being shocked and I was really enthused at how much of the overwhelming majority we had… actually the juniors, it felt like we were taking a bit of control” (P8, resident doctor, striker).* (P8, resident doctor, striker).

#### 3.1.8. Patient Perceptions and Impact

Perceptions on how the strikes impacted patients varied as much as staff experiences of the strikes did. Some participants were certain that the strikes had changed the standard of care offered to patients. Others felt that the strikes, however, had little impact on the provision of care. Some felt that the care provided throughout the strikes could not be differentiated between times when the NHS faced other pressures, like the increased number of admissions and acuity of patients throughout winter. Others also believed that the strikes did not change decision‐making in relation to patient care, noting that when working in A&E regardless of whether there is a strike or not, *“you’re always trying to think about flow and about discharge. So I think you’re always trying to drive to get people through”* (P2, ACP, nonstriker).

In terms of patient perception, participants who went on strike said they received support from patients in regard to the strikes. At least one participant reported frustration from patients, while others felt there was little difference when it came to patient frustration in regard to the strikes, with one participant noting that there was not, *“anyone that was any more disgruntled than normal”* (P2, ACP, nonstriker). Like those above, a number of participants reflected on the fact that many patients were actually unaware that there was a strike.

#### 3.1.9. Views of the NHS

The final theme that emerged related to participants’ views and attitudes towards the NHS. On this, regardless of participants’ profession, whether they went on strike or their seniority, there was substantial convergence when it came to views of the NHS. Many felt that it was a system in crisis that required drastic intervention and a change in course. Almost all expressed a sense of pride and duty working in the system, with most worried about its future. Three subthemes emerged: ‘a system in crisis’, ‘responsibility for the NHS’ and ‘pride and duty’. Each will be discussed below.

#### 3.1.10. A System in Crisis

One point where there was broad agreement was the fact that the NHS was in trouble that it was a system in crisis that needed some type of drastic intervention. A number of participants shared the sentiment that the NHS was *“not the same NHS that I worked in 10 years ago”* (P8, resident doctor, striker), while others reflected on the deeper structural issues that had led to the present crisis. The specific reasons as to why participants believed the system was failing varied. Participants cited underfunding, however, acknowledged that it was often more complicated than this:*“it’s easy to say oh we need more funding, we need more staffing, we need more infrastructure, but how easy is it to actually get that money?”* (P1, PA, nonstriker). Others felt that the NHS was also underappreciated. One participant reflected:

*“I′d like to think that the NHS is built on people that care. Sometimes I feel that a lot of that has been lost, particularly since COVID, but I feel like it′s something that′s there for everyone, no matter what your background, its there to help you. And I think people forget that a lot”* (P2, ACP, non‐striker).

Others pointed to the fact that there was a need for a broader focus, not just on the NHS but also on preventative and social care. Some pointed to failures in leadership, and the fact is that the current issues often go unchallenged:

*“… there′s a lot of, feeling that hospitals are just sticking plaster around the edges, but they [NHS leadership/management] don′t want to admit how bad things are because that makes them look bad in the grander scheme and then they get penalised”* (P8, resident doctor, striker).

Many felt that there was no easy solution, recognising that whatever the solution, substantive change was needed. One participant reflected when considering what was needed: *“I don’t now exactly what the answer is, but something quite dramatic needs to change for us to be able to provide good care for patients*” (P5, resident doctor, nonstriker). Closely related to this and another point of convergence, all participants had substantial concerns for the NHS’s future. That is, while all felt having a system that was free at the point of delivery was the best model of care, all expressed concern about the trajectory the NHS was on.

#### 3.1.11. Responsibility for the NHS

As well as speaking about the issues facing the NHS, participants also spoke about who was primarily responsible for these issues and for the ongoing strikes. Some participants felt that responsibility for the current dispute rested with a number of parties, like the government, the BMA and ultimately heath workers. A number of other participants, however, identified the government as primarily responsible, noting that they have primarily been responsible for conditions that have prompted people to strike. One participant noted that *“they’re the ones who have set up [these] circumstances in the first place. They are to blame for the need to strike”* (P3, resident doctor, striker). While some participants felt that this was a partisan issue, others did not, and some saw this as a broader issue, expressing little faith in any political party to be able to address the problems facing the NHS.

#### 3.1.12. Pride and Duty

Another area where there was substantial convergence related to the sense of pride and or duty that participants had when it came to working in the NHS. One participant reflected on the fact that they felt the NHS was something to be proud of, because it provided care to all; it was a ‘leveller’ in that the entire population had access to care, regardless of their circumstances. Others reflected on their own and personal connections to the NHS. Many saw the NHS as something much bigger than a healthcare system. One participant reflected on the fact that the NHS *“stops us from being a free-for-all individualistic, me me me society, it is the one thing we have left”* (P7, consultant, nonstriker).

While there was substantial pride and a sense of duty for all participants, one participant felt that this also meant there was a duty to work through the current difficulties, to make the system better, they recognised that *“we know we need more resources, but we’re making the best of what we have”* (P4, consultant, nonstriker). At the same time, this participant questioned the commitment of others, particularly those who had spoken out critically about the NHS. This difference perhaps speaks to broader conversations about what it means to value the NHS and what it means in British society, something which will be touched upon below.

## 4. Discussion

This study sought to explore the perspectives of health workers who did and did not go on strike in the United Kingdom throughout the 2022–2024 disputes. The results of this study suggest that there were a number of factors that shaped participants views about these disputes and the NHS more generally. There was convergence on a number of points. Most participants, even those who did not strike, had some sympathy for the strikes and strikers, with all reflecting on the current difficulties facing the NHS. All had carefully considered the pros and cons of (not) striking, with this often coming down to beliefs about the strikes, their demands and how they were conducted. The impact of the strikes was not felt equally. Some felt that any disruption caused by the strikes was not any worse than what would be expected from a normal busy day in the NHS. Others, however, felt the impact of the strikes more acutely. While no clinicians who worked in outpatient services were interviewed, a number of participants identified that these services were particularly impacted. In addition to leading to substantial changes in how care was delivered, the strikes also prompted conflict and solidarity amongst staff. Finally, one point where there was substantial convergence related to participants’ views of the NHS. All saw the NHS as facing multiple and broad crises. At the same time, many felt a sense of pride working within the NHS and the need for better healthcare provision. While there was substantial convergence here, there were points of departure, notably in discussing what should be done about the NHS and what it meant to value the NHS.

The above results speak to the complex and nuanced ways in which health workers in the United Kingdom viewed the recent industrial disputes. They show that support for the strikes, how they were experienced and managed all varied substantially. These findings are consistent with the literature on strikes that show there are often a complex range of factors that dictate whether people support strikes or not, coming down to the strike itself, social, political and historical factors [12] and even individual and personal reasons [7]. Even amongst the relatively small sample above, a range of factors, including position, speciality and seniority, were all influential in dictating experiences and roles in managing care during the strikes. Another thing that stood out in the above results that was discussed explicitly or implied by almost every participant was how disorder and chaos had become normalised within the NHS. For many, they put this down to why they did not acutely feel the impact of the strikes. One finding above that sits comfortably with the existing literature relates to the increased efficiency during the resident doctors’ strikes. [13–16]. This of course is readily explained by the fact that more senior staff is providing frontline care during these periods.

These findings pose a challenge to the current literature, particularly the quantitative literature. These results, along with the results of many other qualitative studies, speak to the varied ways in which the strike was felt for staff and patients. The vast majority of the quantitative literature overlooks these important differences, particularly when looking at patient outcomes and healthcare delivery. Future research should consider the merits of mixed‐methods designs or in trying to capture more granular data [5], to not only capture the overall impact of strike action, but how expereinces differ between staff and patient groups. On this point and looking toward future research, there are a number of limitations that are worth mentioning here. Perhaps most pressingly, the above findings are based on a relatively small sample, with only two people in this group who went on strike. Participants were also recruited via the authors’ personal networks, which could have influenced the above findings. We did not expect or plan to have generalisable findings from this study; however, it is worth keeping in mind that the above themes and issues that were discovered may not be exhaustive. In coming months and years, there will likely be other studies that contextualise and complement our findings; the above findings should be read with this in mind.

In any given year, there are thousands of protests by health workers, many of which are strikes [17]. While strikes reflect the broader forces and pressures that global healthcare systems are under, strike action varies substantially. The findings of this serve as a reminder that beyond simplified metrics of disruption or delay, there are deeply human factors—ethical, social and professional—that shape attitudes toward and experiences of industrial action.

## Author Contributions

Ryan Essex and David Smithard conceptualised the study. Ryan Essex led data collection and analysis, supported by David Smithard. Ryan Essex led the initial write up of the paper, supported by David Smithard in subsequent revisions.

## Funding

No funding was received for this submission. Open access publishing facilitated by University of New South Wales, as part of the Wiley ‐ University of New South Wales agreement via the Council of Australasian University Librarians.

## Ethics Statement

This study was approved by the University of Greenwich Research Ethics Committee. All participants provided written informed consent prior to participating in this study.

## Conflicts of Interest

The authors declare no conflicts of interest.

## Endnotes

^1^Throughout this paper, we use the term industrial action/dispute as an umbrella term to refer to strikes, but also other actions and the negotiation that surrounds such action. We refer to strikes and strike action as they are typically understood, to mean the temporary stoppage of work, associated with some form of demand of grievance.

^2^Please note—as participants were recruited outside of the NHS and as interviews were conducted outside of work hours/premises, NHS ethics approval was not required.

## Supporting Information

Supporting Information includes an interview schedule that was used to guide interviews.

## Supporting information


**Supporting Information** Additional supporting information can be found online in the Supporting Information section.

## Data Availability

The data that support the findings of this study are available on request from the corresponding author. The data are not publicly available due to privacy or ethical restrictions.
