# Supplementary material for: UK Health Workers’ Experiences of Striking and Not Striking in the 2022–2024 Industrial Disputes
Source: J Nurs Manag. 2026 Feb 26;2026:3663164. doi: 10.1155/jonm/3663164 (PMC12936980; doi:10.1155/jonm/3663164)
Supplement: Supplementary file 1 — Supporting Information Additional supporting information can be found online in the Supporting Information section. [file JONM-2026-3663164-s001.docx]

Could you please tell me what profession or registration you hold?

How long have you worked in the NHS or held your position?

What does the NHS mean to you? Does it live up to its expectations? If not, what should it be?

Have you been on strike? Or worked throughout the strikes?

Can you tell me what steps were taken by management and/or your trust to mitigate the impact of the strikes?

**For people who worked through the strikes**

Can you tell me about working through the strikes? Did it change your role for better or worse?

Did it change how people made clinical decisions?

Were there any differences when doctors or nurses went on strike?

**For all participants**

Have the strikes caused any conflicts or difficulties?

Has there been any animosity to strikers/non-strikers?

Have you noticed the impact of the strikes on patients?

Has strike action impacted your relationship with your colleagues? Has it impacted teamwork or broader solidarity?

Who do you feel is responsible for the strikes and/or the broader difficulties the NHS is facing?

How do you feel about the future of the NHS and the current unrest amongst staff?

Is there any other information you would like to share?
